# Association Between ST-Segment Deviation in Electrocardiography and 30-Day Mortality in Non-Cardiac Critically Ill Patients: A Retrospective Single-Center Study

**DOI:** 10.3390/jcm14144911

**Published:** 2025-07-10

**Authors:** Rafał Świstek, Emil Dadański, Aleksandra Kurzyca, Jakub Droś, Patryk Kasongo, Jakub Konieczyński, Joanna Jóźwik, Tomasz Drygalski, Michał Terlecki

**Affiliations:** 1 Department of Interdisciplinary Intensive Care, Jagiellonian University Medical College, 30-688 Krakow, Poland; rswistek@gmail.com (R.Ś.); jakub.dros@gmail.com (J.D.); patrykkasongo@gmail.com (P.K.); konieczynski.jakub@gmail.com (J.K.); dtomec@gmail.com (T.D.); 2 First Department of Cardiology, Interventional Electrocardiology and Arterial Hypertension, Jagiellonian University Medical College, 31-902 Krakow, Poland; emil.dadanski@gmail.com (E.D.); olakurzyca@gmail.com (A.K.); 3 Student’s Scientific Group in the Department of Interdisciplinary Intensive Care, Jagiellonian University Medical College, 31-902 Krakow, Poland; j.jozwik@student.uj.edu.pl

**Keywords:** ECG, ST deviation, QT interval, QRS prolongation, SOFA score, critical care

## Abstract

**Background/Objectives**: ST-segment deviation (STD) on electrocardiography (ECG) may reflect myocardial injury in critically ill patients. However, its prognostic significance in non-cardiac intensive care unit (ICU) populations remains unclear. We aimed to assess the association between STD on ICU admission and 30-day mortality and to evaluate its incremental prognostic value beyond the SOFA score. **Methods:** In this retrospective single-center study, we included 307 consecutive ICU patients (median age: 64.0 years; 65.5% men). Patients with acute cardiac conditions were excluded. STD was defined as ≥1 mm ST elevation or depression in any lead on standard 12-lead ECG performed on admission. The primary outcome was 30-day all-cause mortality. Prognostic associations were assessed using multivariable Cox regression adjusted for SOFA score. Discriminative performance was evaluated by comparing ROC curves for models with and without STD, with bootstrap-based testing (1000 iterations) to assess significance. **Results:** STD was present in 126 patients (41.0%) and occurred more frequently in non-survivors (47.6% vs. 36.5%, *p* = 0.033. In Cox regression, STD was independently associated with 30-day mortality (HR = 1.534; 95% CI: 1.081–2.177; *p* = 0.017), even after adjustment for SOFA score. This association remained statistically robust in bootstrap validation. The addition of STD amplitude to the SOFA score modestly improved model discrimination with a borderline significant difference between the areas under the curve (ΔAUC = 0.005, *p* = 0.0581). **Conclusions:** ST-segment deviation on the admission ECG is an independent predictor of 30-day mortality in non-cardiac critically ill patients and may enhance risk stratification beyond the SOFA score.

## 1. Introduction

The high mortality rate among patients requiring intensive therapy unit (ICU) hospitalization should be taken into consideration and an adequate assessment of the degree of individual organ failure and prognosis evaluation in this group of patients is of crucial importance in terms of implementing appropriate management strategies and assessing the effectiveness of treatment. The sequential organ failure assessment (SOFA) score is one of the most common scales used in the intensive care unit (ICU) to evaluate the degree of individual organ failure [1]. The SOFA score has been validated for assessing organ dysfunction in many clinical scenarios including medical, traumatic, surgical, cardiac, neurological, transplant, respiratory care and step-down units [2]. Originally, the SOFA score consisted of the evaluation of six organ systems: respiratory, cardiovascular, neurological, hepatic, renal and coagulation [1,2,3]. Even though the SOFA score was originally designed for the assessment of organ dysfunction, it has been repeatedly shown that the SOFA score also correlates in critically ill patients with survival [2]. However, the rapid advancement in critical care medicine has resulted in an increasing number of reports suggesting that the prognostic value of the SOFA score may be unsatisfactory [1] and most doubts concern the proper evaluation of the degree of dysfunction of the cardiovascular system, e.g., currently in clinical practice, we use vasopressors that are not included in the SOFA score (e.g., vasopressin), and drugs previously routinely used and included in the SOFA (dopamine) are currently used incidentally [4,5]. Hence, there is a need to look for additional tools that, in addition to the parameters included in the SOFA score, will allow for a better evaluation of the patient’s clinical condition and will provide additional prognostic value.

Standard 12-lead electrocardiography (ECG) is a widely known diagnostic tool, available in every ICU ward, and it has been clearly shown that ECG analysis can provide prognostic insights, particularly in patients with cardiovascular diseases [6,7]. Even after the exclusion of acute cardiac patients, myocardial dysfunction and injury are often seen among the critically ill. Among various ECG abnormalities, ST-segment deviation (STD)—defined as ST elevation or depression—may reflect myocardial ischemia or injury, conditions known to be associated with poor outcomes in the critically ill [8]. Despite its potential relevance, STD is not currently included in standard scoring systems used to assess organ dysfunction in ICU settings, such as the SOFA score. Furthermore, the frequency and prognostic relevance of STD specifically in non-cardiac ICU populations have not been well characterized, and it remains unclear whether STD provides independent prognostic value beyond established clinical tools.

Therefore, we aimed to assess the prevalence of ST-segment deviation on admission ECGs in non-cardiac critically ill patients and evaluate its association with 30-day mortality. We specifically hypothesized that STD would provide independent prognostic information and improve risk stratification beyond the SOFA score.

## 2. Materials and Methods

### 2.1. Data Collection and Study Endpoint

**Figure 1:**
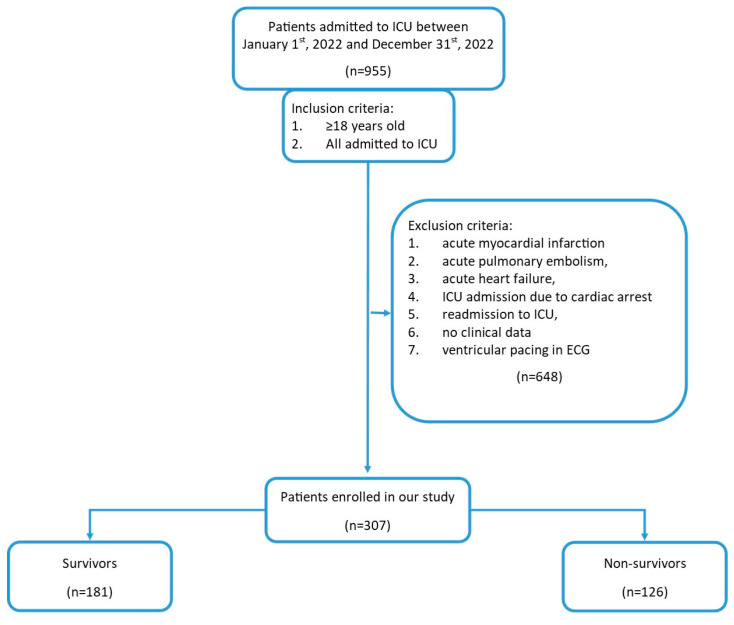
Flowchart of the study. Abbreviations: ICU—intensive care unit, ECG—electrocardiogram.

We retrospectively analyzed the medical records of unselected, consecutive patients admitted to the intensive care unit in University Hospital, Krakow, Poland, between January 2022 and December 2022, who fulfilled the inclusion criteria and did not meet the exclusion criteria. All patients who were admitted to the ICU due to acute cardiac disease (i.e., acute myocardial infarction, acute heart failure, acute pulmonary embolism, cardiac arrest) during the analyzed period were excluded from the study and the final study population consisted exclusively of non-cardiac critically ill patients (Figure 1). The study was designed and reported in accordance with the STROBE (strengthening the reporting of observational studies in epidemiology) guidelines for observational cohort studies (Supplementary Materials Table S1) [9].

A standard 12-lead ECG was performed in all patients immediately upon admission to the ICU, with recordings obtained in the supine position using a Mortara ELI 280 device (Manufactured by Mortara Instrument, Inc., Milwaukee, WI, USA) (amplitude: 10 mm/mV; speed: 25 mm/s). All ECGs were independently reviewed by two experienced cardiologists blinded to clinical information and patient outcomes. ST-segment deviation (STD) was defined as ≥1 mm elevation or depression measured between the J point and the PR-segment baseline in at least one lead. This definition followed the criteria used in the GRACE risk score for acute cardiac patients and the Fourth Universal Definition of Myocardial Infarction [10,11,12,13]. Specifically, STD was considered present when there was a horizontal or down sloping ST depression ≥1 mm (0.1 mV) in at least one lead and/or ST elevation ≥1 mm in any lead other than V2–V3; for leads V2–V3, the cutoff for ST elevation was ≥2 mm in men ≥40 years, ≥2.5 mm in men <40 years, or ≥1.5 mm in women. The magnitude of deviation was measured at 60 ms after the J point (J + 60 ms), relative to the isoelectric PR segment. For each patient with ST-segment deviation, the sum of ST-segment deviation amplitudes across all leads (in millimeters) was calculated as the sum of absolute ST elevations and depressions across all 12 ECG leads on admission.

Clinical data including demographics, comorbidities, in-hospital course, laboratory findings, and outcomes were extracted from the electronic medical records used at the University Hospital. The SOFA score was calculated based on clinical and laboratory data from the first 24 h of ICU stay. The primary outcome was all-cause mortality within 30 days of ICU admission. Information on survival status was obtained from the National Electronic Population Registration System of Poland.

### 2.2. Statistical Analysis

Categorical variables are summarized as absolute values and percentages, while continuous variables are presented as means with standard deviations (SD) or medians with interquartile ranges (IQR), depending on data distribution. The Shapiro–Wilk test was used to assess normality, and the Levene test was used to evaluate homogeneity of variances. Normally distributed continuous variables were compared using Student’s *t*-test or Welch’s *t*-test, depending on variance equality. Non-normally distributed variables were analyzed using the Mann–Whitney U-test. Ordinal variables were assessed using the Cochran–Armitage test for trend, and categorical variables were compared using Pearson’s chi-squared test or Fisher’s exact test (Monte Carlo simulation applied when >20% of expected frequencies were <5).

The primary outcome was all-cause 30-day mortality. The prognostic value of ST-segment deviation (STD) for 30-day mortality was first assessed using univariable Cox proportional hazards regression. Subsequently, a multivariable model was constructed adjusting for the SOFA score to evaluate whether STD remained independently associated with the outcome. Hazard ratios (HR) with 95% confidence intervals (CI) were calculated for both models. To validate the robustness of the multivariable model, a bootstrap resampling procedure with 1000 iterations was performed using the bias-corrected and accelerated (BCa) method to generate confidence intervals for regression coefficients and *p*-values. The proportional hazards assumption was assessed with Schoenfeld residuals and graphical diagnostics.

Additionally, receiver operating characteristic (ROC) analysis was performed to evaluate model discrimination. The area under the curve (AUC) for the model including the SOFA score alone was compared to that of the model incorporating both the SOFA score and the total amplitude of ST-segment deviation. The difference in AUCs was assessed using DeLong’s test for correlated ROC curves, with statistical significance defined as *p* < 0.05 [14].

All analyses were performed using IBM SPSS Statistics version 18.0 (IBM Corp., Chicago, IL, USA), R software version 3.4.1 (R Foundation for Statistical Computing, Vienna, Austria), and G*Power version 3.1.9.7. A priori power analysis was performed to assess the ability to detect the observed difference in STD prevalence between survivors and non-survivors. Assuming equal group sizes, the power to detect this difference at a two-sided alpha level of 0.05 equaled 80%.

The study was conducted following the guidelines of the Declaration of Helsinki and was approved by the Bioethics Committee of Jagiellonian University, decision number 118.0043.1.177.2025.

## 3. Results

### 3.1. Clinical Characteristics of Study Subjects

Our study group consisted of 307 patients (65.5% men). The median (IQR) age was 64.0 (50.0–71.0) years. The median (IQR) SOFA score was 10.0 (8.0–12.0). More than half of the patients were admitted with surgical diagnoses (*n* = 174; 56.68%) while (*n* = 133; 43.32%) were admitted with medical ones. Of the participants, 48.9% had arterial hypertension, 22.8% had diabetes, 15.6% had a history of stable ischemic heart disease, and 9.8% had a history of previous myocardial infarction. There were 59% (*n* = 181) survivors and 41% (*n* = 126) non-survivors in the analyzed group. The median [interquartile range (IQR)] length of ICU stay was 11 (3–21) days. In the entire study cohort, the STD on the admission ECG was observed in 126 patients (41.0%). The median sum of ST-segment deviation amplitudes across all leads was 0.0 mm (IQR: 0.0–2.5 mm) in the entire study group (Table 1 and Table 2).

### 3.2. Prognostic Significance of ECG Abnormalities on Admission to ICU in Relation to 30-Day Mortality

**Figure 2:**
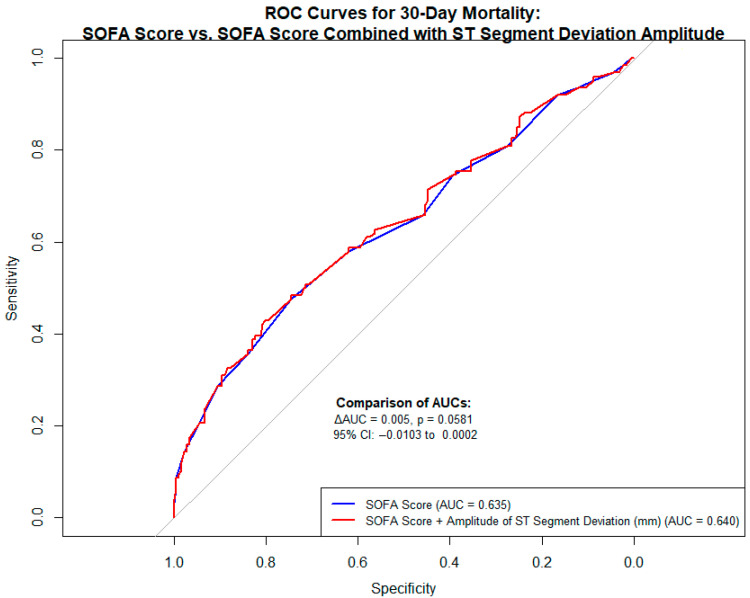
Receiver operating characteristic (ROC) curves comparing the performance of the SOFA score alone and the SOFA score combined with the amplitude of ST-segment deviation on admission ECG in predicting 30-day mortality.

We observed a significant difference in the frequency rate of STD among non-survivors in comparison to survivors. In the Cox regression, the STD was an independent prognostic factor for 30-day mortality in univariable analysis (HR = 1.512; 95% CI: 1.065–2.145; *p* = 0.021) and remained significant even after adjustment for SOFA score (HR = 1.534; 95% CI: 1.081–2.177; *p* = 0.017) (Table 3). To ensure the robustness of this result, a bootstrap analysis with 1000 resamples was performed, confirming statistical significance (bootstrap 95% BCa CI for β coefficient: 0.012–0.851; *p* = 0.020). In receiver operating characteristic (ROC) analysis, the addition of STD to the SOFA score slightly improved model discrimination for predicting 30-day mortality (AUC = 0.640 vs. 0.635), with a borderline significant difference between the areas under the curve (ΔAUC = 0.005, *p* = 0.0581; 95% CI: CI: −0.010 to 0.000; DeLong test) (Figure 2).

## 4. Discussion

Our study in critically ill non-cardiac patients confirms that the occurrence of STD on admission ECG was frequently observed and it is related to lower survival probability in 30-day follow-up, even after adjustment for SOFA score. Consistently, ROC analysis suggested a potential trend toward improved model discrimination with the inclusion of STD amplitude. Therefore, this ECG parameter may serve as a valuable adjunct for early risk stratification in critically ill non-cardiac patients.

Numerous pathophysiologic reasons are causing cardiac dysfunction among critically ill patients and, in many clinical scenarios, myocardial injury has been proven to be associated with poor prognosis in this group of patients [15,16]. It is therefore not surprising that the appropriate evaluation of the advancement of cardiac failure in critically ill patients is necessary. Among different stratification tools in the ICU, the SOFA score remains the most commonly used scale for evaluating multiple organ failure which correlates with mortality risk [2,3]. However, many studies indicate that assessing the degree of advancement in myocardial dysfunction using the SOFA Score is insufficient [1,5,17].

It has been repeatedly proven that ECG is a study that provides plenty of valuable information on the status of the myocardium. Numerous pathologic ECG signs of prognostic importance can be observed among critically ill patients even after the exclusion of patients admitted due to acute cardiac conditions [18]. However, none of the stratification tools, including the SOFA score, take into account any ECG parameters in their assessment.

The 12-lead ECG is a valuable diagnostic tool with several parameters demonstrating substantial prognostic significance in the general population. Among them, ST segment deviation (STD) has been consistently identified as an important predictor of adverse outcomes., but the data on the incidence of this ECG pattern among critically ill patients are less numerous and are isolated among the non-cardiac critically ill. However, numerous mechanisms may lead to cardiac dysfunction in critically ill patients, which may give a picture of the above-mentioned pattern in ECG [7].

Our findings are consistent with the observations by Russo et al., who highlighted the high prevalence of electrocardiographic abnormalities in ICU patients [19]. In our study we showed that the incidence of STD was quite high among critically ill patients, and additionally, it turned out to be a significant parameter associated with increased mortality. To further validate the robustness of our findings, we performed a bootstrap analysis with 1000 resamples using the bias-corrected and accelerated (BCa) method. The results confirmed the statistical significance of ST-segment deviation as an independent predictor of 30-day mortality, even after adjustment for the SOFA score. This reinforces the reliability of our model and supports the prognostic role of STD in this clinical setting. The above observations have their pathophysiological justification. Myocardial injury and myocardial ischemia seem to be among the most important clinical conditions that are associated with unfavorable outcomes in critically ill patients [20]. Electrophysiologically, ST-segment deviation in ECG is explained as the difference in electrical potential between ischemic and normal myocardium, leading to the ST segment’s displacement (upward or downward). In critically ill patients, STD should, therefore, be viewed as an additional indicator of myocardial oxygen supply–demand mismatch, which may reflect the severity of metabolic insufficiency and the grade of multiple organ failure [21,22]. STD observed in ECG, in the group of patients analyzed by us, can therefore be interpreted as a sign of ongoing myocardial injury [20,21,22], irrespective of the main condition leading to the need for hospitalization in the ICU.

Being skeptical about the prognostic value of the ECG in the ICU, one may suggest that in this group of patients, relying on the measurement of laboratory markers of myocardial necrosis (troponin) may be more efficient because their elevation justifies the diagnosis of myocardial injury in critically ill patients [23]. Of course, it is well known that myocardial injury is a significant clinical problem among critically ill patients and is related to increased mortality. In our study, similar to other observational studies, the percentage of patients with elevated troponins was high, and there is no doubt that elevated values of myocardial necrosis markers also have prognostic value [24,25]. However, the differentiation between injury and MI remains challenging in critically ill patients and it seems that, in routine practice, troponin measurements may often add both clarity and confusion to the physicians simultaneously, thus we believe that the results of our study will encourage physicians to make a more thorough interpretation of the ECG in this group of patients because it provides a wider range of information (than troponin measurement alone) on the ongoing myocardial injury, and only the combined interpretation of both ECG pathologies and troponin values (in addition to the whole clinical picture) increases the possibility of making the correct diagnosis with an adequate differentiation between myocardial injury and MI [25]. It should be emphasized that the problem is of great clinical relevance because myocardial injury is a common event in patients hospitalized in an ICU (even reaching up to 40%) and post-mortem examinations among critically ill patients suggest that MI is among the most frequently missed diagnoses likely to impact outcome [26,27].

An additional point of discussion concerns the comparative analysis of ROCs. While the improvement in model discrimination after adding STD amplitude to the SOFA score did not reach conventional statistical significance, a trend toward better prognostic performance was observed. This suggests that the inclusion of STD amplitude may offer incremental prognostic value beyond the SOFA score alone. Although this finding requires validation in larger cohorts, the observed trend additionally supports our hypothesis that STD could contribute to early risk stratification in non-cardiac critically ill patients. However, it should be clearly emphasized that external validation in independent multicenter datasets is essential to confirm the reproducibility and generalizability of these findings before any implementation into clinical practice can be recommended.

Our study provides novel and clinically relevant insights, as although the prognostic value of STD has been extensively validated in patients with acute cardiac conditions, there is a notable lack of evidence regarding its applicability and significance in non-cardiac critically ill populations. Our findings confirming the prognostic significance of STD in non-cardiac critically ill patients may indicate that the group of patients who experience the abovementioned ECG changes may require both more advanced clinical evaluation (including serial ECG recordings, troponin measurements, echocardiographic evaluation, etc.) and a more thorough consideration of the indications for using additional therapeutic strategies. Implementation of treatment aimed at reducing myocardial injury could potentially offer a survival benefit for this group of patients and an increasing number of studies indicate that despite how counterintuitive it seems, this group of patients might benefit, especially from cardioselective beta-blockers. Even in the group of patients with advanced multi-organ failure, including those requiring pressor amines, this treatment showed a proven beneficial effect in reducing oxygen demand and reducing myocardial injury, which may turn into improved prognosis in this group of patients [28].

### Limitations

A single-center study is prone to error caused by the profile of the department’s activity, the qualification of patients with identical environmental conditions, and the adopted procedure. However, due to the multi-profile nature of our department, it was possible to study the analyzed ECG parameter in a mixed population (including surgical, neurosurgical, general, septic, etc.). Another limitation of our work is the fact that only ECG on admission was available for us to analyze; thus, follow-up in the following days and analysis of the occurring changes was impossible. In this respect, further research and assessment of the possible impact of newly emerging changes are necessary. Another limitation of the study is the size of the study group, but it was statistically found to be representative of the assessed parameters. The power analysis was based on an assumption of equal group sizes, which may have slightly overestimated the actual power given the unbalanced number of survivors and non-survivors. Nevertheless, the calculated power equaled 80% which suggests that the sample was reasonably adequate to detect the observed difference in STD prevalence. Although the sample size was moderate, it was deemed statistically adequate for the associations investigated. Due to the limited number of predictors, we did not employ more advanced statistical approaches such as LASSO regression or AIC-based model selection. Our main objective was to determine whether ST-segment deviation provides additive prognostic information beyond the SOFA score. In this context, the use of multivariable regression and ROC-based comparison between models—with and without STD—was considered the most appropriate and clinically interpretable analytical strategy. Finally, external validation was not feasible within the scope of this study. To reduce the risk of overfitting and to enhance internal validity, we applied bootstrap resampling (1000 iterations), which confirmed the robustness of our findings. Future studies involving larger and multicenter cohorts should aim to externally validate these results and may benefit from applying penalized regression techniques or information–theoretical model selection methods.

## 5. Conclusions

ST-segment deviation on an admission ECG is an independent predictor of 30-day mortality in non-cardiac critically ill patients and may enhance risk stratification beyond the SOFA score.

## Figures and Tables

**Table 1:** Baseline clinical characteristics according to 30-day ICU survival status.

Parameter	Survivors (*n* = 181; 59.0%)	Non-Survivors (*n* = 126; 41.0%)	*p* Value
Age [years], median, (IQR)	63.0 (47.0–69.5)	67.0 (55.0–73.0)	0.040
Male, *n*, (%)	118 (65.2)	83 (65.9)	0.500
BMI ^a^ [kg/m^2^], mean, (SD)	27.61 (6.53)	28.31 (6.23)	0.351
Diabetes mellitus, *n* (%)	38 (21.0)	32 (25.4)	0.221
Arterial hypertension, *n* (%)	83 (45.9)	67 (53.2)	0.126
COPD, *n* (%)	12 (6.6)	12 (9.5)	0.237
Ischemic heart disease, *n* (%)	18 (9.9)	30 (23.8)	0.001
Chronic kidney disease, *n* (%)	16 (8.8)	18 (14.3)	0.096
History of myocardial infarction, *n* (%)	12 (6.6)	18 (14.3)	0.022
Chronic hepatic failure, *n* (%)	2 (1.1)	14 (11.1)	<0.001
Heart failure, *n* (%)	22 (12.2)	23 (18.3)	0.094
Active malignancy, *n* (%)	17 (9.4)	11 (8.7)	0.505
History of acute ischemic stroke, *n* (%)	6 (3.3)	7 (5.6)	0.249
Admission category			
Medical, *n* (%)	92 (50.8)	41 (32.5)	0.001
Surgical, *n* (%)	89 (49.2)	85 (67.5)	0.001

Abbreviations: BMI—body mass index; COPD—chronic obstructive pulmonary disease; IQR—interquartile range; ^a^ Data available in 300 patients for BMI.

**Table 2:** Clinical, laboratory and ECG parameters on admission to ICU and the outcomes according to 30-day ICU survival status.

Parameter	Survivors (*n* = 181; 59.0%)	Non-Survivors (*n* = 126; 41.0%)	*p* Value
SOFA, median (IQR)	10.0 (7.0–12.0)	11.0 (8.0–14.0)	<0.001
Sinus rhythm in ECG on admission, *n* (%)	154 (85.1)	99 (78.6)	0.094
Atrial fibrillation in ECG on admission, *n* (IQR)	21 (11.6)	17 (13.5)	0.373
ST-segment deviation, *n* (IQR)	66 (36.5)	60 (47.6)	0.033
Amplitude of ST-segment deviation [mm], median (IQR)	0.0 (0.0–2.5)	0.0 (0.0–3.0)	0.120
Hospital length of stay [days], median (IQR)	29.0 (17.0–45.0)	10.0 (4.0–16.0)	<0.001
ICU length of stay[days], median (IQR)	14.0 (5.5–32.0)	7.0 (2.0–14.3)	<0.001
Troponin[ng/mL], median (IQR)	55.9 (20.9–143.9)	82.6 (22.6–536.3)	0.025
NT-pro-BNP ^a^ [pg/mL], median (IQR)	1337.5 (386.5–3473.0)	2365.0 (553.5–8861.0)	0.011
K + [mmol/L], median (IQR)	4.1 (3.7–4.5)	4.20 (3.80–5.0)	0.039
Na + [mmol/L], median (IQR)	139.0 (136.0–141.0)	138.0 (135.0–141.0	0.727
Anion gap ^a^ on admission [mmol/L], median (IQR)	10.7 (7.95–13.2)	12.0 (8.5–16.9)	0.007
Base excess on admission [mmol/L], median (IQR)	−3.8 (−6.9–−0.5)	−6.4 (−12.6–−1.3)	<0.001
pH on admission, median (IQR)	7.309 (7.236–7.367)	7.295 (7.117–7.365)	0.005
Lactates on admission [mmol/L], median (IQR)	1.9 (1.1–3.2)	3.2 (1.5–8.5)	<0.001
Mean arterial pressure on admission [mmHg], mean (SD)	78.2 (21.6)	72.1 (22.9)	0.018
Heart rate on admission [bpm], mean (SD)	89.8 (24.9)	93.2 (24.9)	0.239
Mean noradrenaline dose on admission [ucg/kg/min], median (IQR)	0.1 (0.02–0.20)	0.10 (0.04–0.40)	0.040
Mechanical ventilation on admission, *n* (%)	160 (58.4)	114 (41.6)	0.351

Abbreviations: SOFA—sequential organ failure assessment, BE—base excess; bpm—beats per minute; ICU—intensive care unit, IQR—interquartile range, SD—standard deviation, CI—confidence interval. ^a^ Data available in 303 patients for anion gap and in 291 patients for NT-pro-BNP.

**Table 3:** Univariable and multivariable Cox regression model assessing the risk of death at a 30-day follow-up (model included ECG parameters and SOFA score on admission).

Variable	Univariable Analysis		Multivariable Analysis			
	HR (95% CI)	*p* Value	HR (95% CI)	*p* Value	Bootstrap 95% CI (BCa)	Bootstrap *p* Value
ST-segment deviation	1.512 (1.065–2.145)	0.021	1.534 (1.081–2.177)	0.017	1.013–2.342	0.020
SOFA score	1.137 (1.082–1.194)	<0.001	1.139 (1.083–1.197)	<0.001	1.083–1.239	<0.001

Abbreviations: CI—confidence interval, HR—hazard ratio, SOFA score—sequential organ failure assessment. Bootstrap confidence intervals were derived using 1000 resamples and the bias-corrected and accelerated (BCa) method.

## Data Availability

The data presented in this study are available on request from the corresponding author.

## Supplementary Materials

The following supporting information can be downloaded at: https://www.mdpi.com/article/10.3390/jcm14144911/s1, Table S1: STROBE Statement—Checklist of items that should be included in reports of cohort studies.

## Abbreviations

The following abbreviations are used in this manuscript:

AUC	area under the curve
BE	base excess
BMI	body mass index
BNP	B-type natriuretic peptide
CI	confidence interval
COPD	chronic obstructive pulmonary disease
ECG	electrocardiogram
HR	hazard ratio
ICU	intensive care unit
IQR	interquartile range
MI	myocardial infarction
NT	N-terminal
ROC	receiver operating characteristic
SD	standard deviation
SOFA	sequential organ failure assessment
STD	ST-segment deviation
