# Supplementary material for: Association Between ST-Segment Deviation in Electrocardiography and 30-Day Mortality in Non-Cardiac Critically Ill Patients: A Retrospective Single-Center Study
Source: J Clin Med. 2025 Jul 10;14(14):4911. doi: 10.3390/jcm14144911 (PMC12295174; doi:10.3390/jcm14144911)
Supplement: Supplementary file 1 [file jcm-14-04911-s001.zip › jcm-3717394-supplementary.pdf]

Table S1. STROBE Statement—Checklist of items that should be included in reports of ***cohort studies***

	Item No	Recommendation	Lines in manuscript
**Title and abstract**	1	(*a*) Indicate the study's design with a commonly used term in the title or the abstract	indicated in the title
		(*b*) Provide in the abstract an informative and balanced summary of what was done and what was found	The abstract includes a structured summary of the background, methods, results and conclusion
**Introduction**			
Background/rationale	2	Explain the scientific background and rationale for the investigation being reported	provided in the Introduction (lines 40–72).
Objectives	3	State specific objectives, including any prespecified hypotheses	last paragraph of the Introduction (lines 74–47).
**Methods**			
Study design	4	Present key elements of study design early in the paper	described early in the Methods section (lines 78–88).
Setting	5	Describe the setting, locations, and relevant dates, including periods of recruitment, exposure, follow-up, and data collection	Study setting and time frame (University Hospital ICU, Krakow, Jan–Dec 2022) are detailed in lines 80–82.
Participants	6	(*a*) Give the eligibility criteria, and the sources and methods of selection of participants. Describe methods of follow-up	Inclusion/exclusion criteria and data source are detailed in lines 82–86
		(*b*) For matched studies, give matching criteria and number of exposed and unexposed	Not applicable – no matching was performed.
Variables	7	Clearly define all outcomes, exposures, predictors, potential confounders, and effect modifiers. Give diagnostic criteria, if applicable	All variables (exposures, outcomes, confounders) are defined in lines 105–110.
Data sources/ measurement	8*	For each variable of interest, give sources of data and details of methods of assessment (measurement). Describe comparability of assessment methods if there is more than one group	Source and method of measurement of each variable (e.g. ECG, labs, SOFA)

			are provided in lines 89–108.
Bias	9	Describe any efforts to address potential sources of bias	No formal bias analysis, but use of consecutive patients and blinding of ECG assessors mitigates selection bias.
Study size	10	Explain how the study size was arrived at	lines 141–144
Quantitative variables	11	Explain how quantitative variables were handled in the analyses. If applicable, describe which groupings were chosen and why	Quantitative variables are described with methods for transformation and grouping (lines 113–122).
Statistical methods	12	(*a*) Describe all statistical methods, including those used to control for confounding	Statistical methods including confounder adjustment using Cox models and ROC analysis are described in lines 123–138
		(*b*) Describe any methods used to examine subgroups and interactions	No subgroup or interaction analyses were performed.
		(*c*) Explain how missing data were addressed	Missing data handling is noted by footnotes under tables (e.g. NT-proBNP availability); no imputation was performed. Importantly, for all main parameters analysed – 100% of data were available and there were no missing data.
		(*d*) If applicable, explain how loss to follow-up was addressed	Not applicable – 30-day mortality was available for all patients from national registry
		(*e*) Describe any sensitivity analyses	Bootstrap analysis with 1000 samples was used as a sensitivity analysis (lines 128–132).
**Results**			
Participants	13*	(a) Report numbers of individuals at each stage of study—eg numbers potentially eligible,	Number of included patients and flowchart

		examined for eligibility, confirmed eligible, included in the study, completing follow-up, and analysed	are presented in lines 151 and Figure 1.
		(b) Give reasons for non-participation at each stage	Reasons for exclusion are described based on predefined criteria.
		(c) Consider use of a flow diagram	A study flowchart (Figure 1) is included.
Descriptive data	14*	(a) Give characteristics of study participants (eg demographic, clinical, social) and information on exposures and potential confounders	Baseline characteristics and confounders are described in Tables 1 and 2.
		(b) Indicate number of participants with missing data for each variable of interest	Missing data are indicated in table footnotes.
		(c) Summarise follow-up time (eg, average and total amount)	Follow-up time is fixed at 30 days; ICU/hospital stay durations are reported.
Outcome data	15*	Report numbers of outcome events or summary measures over time	Outcome data (mortality) are reported in Tables 2 and 3 and text (lines 156–158).
Main results	16	(*a*) Give unadjusted estimates and, if applicable, confounder-adjusted estimates and their precision (eg, 95% confidence interval). Make clear which confounders were adjusted for and why they were included	Unadjusted and adjusted HRs with 95% CIs are provided in Table 3.
		(*b*) Report category boundaries when continuous variables were categorized	Categories for continuous variables are reported (e.g. age, SOFA).
		(*c*) If relevant, consider translating estimates of relative risk into absolute risk for a meaningful time period	Absolute risk was not calculated;
Other analyses	17	Report other analyses done—eg analyses of subgroups and interactions, and sensitivity analyses	Other analyses include ROC comparison and bootstrap validation (lines 168–174).
**Discussion**			
Key results	18	Summarise key results with reference to study objectives	Key findings are summarized in the first

			paragraph of the Discussion (lines 260–270).
Limitations	19	Discuss limitations of the study, taking into account sources of potential bias or imprecision. Discuss both direction and magnitude of any potential bias	Study limitations are discussed in detail in the Limitations section (lines 321–347).
Interpretation	20	Give a cautious overall interpretation of results considering objectives, limitations, multiplicity of analyses, results from similar studies, and other relevant evidence	Interpretation considers objectives, existing literature, and clinical relevance (lines 233–319).
Generalisability	21	Discuss the generalisability (external validity) of the study results	Generalizability and need for external validation are addressed (lines 301–304).
**Other information**			
Funding	22	Give the source of funding and the role of the funders for the present study and, if applicable, for the original study on which the present article is based	Funding statement included: 'This research received no external funding' (line 366).

*Give information separately for exposed and unexposed groups.

Note: An Explanation and Elaboration article discusses each checklist item and gives methodological background and published examples of transparent reporting. The STROBE checklist is best used in conjunction with this article (freely available on the Web sites of PLoS Medicine at http://www.plosmedicine.org/, Annals of Internal Medicine at http://www.annals.org/, and Epidemiology at http://www.epidem.com/). Information on the STROBE Initiative is available at http://www.strobe-statement.org.
